# Dataset for acrylate/silica nanoparticles formulations and photocured composites: Viscosity, filler dispersion and bulk Poisson׳s ratio

**DOI:** 10.1016/j.dib.2017.04.040

**Published:** 2017-04-28

**Authors:** Hubert Gojzewski, Mariola Sadej, Ewa Andrzejewska, Martyna Kokowska

**Affiliations:** a Institute of Physics, Poznan University of Technology, Piotrowo 3, 60-965 Poznan, Poland; b Department of Theory & Bio-Systems, Max Planck Institute of Colloids and Interfaces, Am Mühlenberg 1 Golm, 14476 Potsdam, Germany; c Faculty of Chemical Technology, Poznan University of Technology, Berdychowo 4, 60-965 Poznan, Poland

## Abstract

UV-curable polymer composites are of importance in industry, biomedical applications, scientific fields, and daily life. Outstanding physical properties of polymer composites were achieved with nanoparticles as filler, primarily in enhancing mechanical strength or barrier properties. Structure-property relationships of the resulting nanocomposites are dictated by the polymer-filler molecular architecture, i.e. interactions between polymer matrix and filler, and high surface area to volume ratio of the filler particles. Among monomers, acrylates and methacrylates attracted wide attention due to their ease of polymerization and excellent physicochemical and mechanical properties of the derived polymers. We prepared and photopolymerized two series of formulations containing hydrophobized silica nanofiller (Aerosil R7200) dispersed in 2-hydroxyethyl acrylate (HEA) or polyethylene glycol diacrylate (PEGDA) monomers. We compared selected physical properties of the formulations, both before and after photocuring; specifically the viscosity of formulations and dispersion of the filler in the polymer matrices. Additionally, we estimated the bulk Poisson׳s ratio of the investigated nanocomposites. This article contains data related to the research article entitled “Nanoscale Young׳s modulus and surface morphology in photocurable polyacrylate/nanosilica composites” (Gojzewski et al., 2017) [1].

**Specifications Table**

**Table:** 

Subject area	Physics, Chemistry
More specific subject area	Photocurable polyacrylate-based composites with silica nanoparticles
Type of data	Table, text file, figures
How data was acquired	Viscometer (model DV-II+ PRO, Brookfield Engineering Laboratories, USA), SEM (model LEO 1530 Gemini, Carl Zeiss NTS, Germany), hydrostatic mass balance (model AD200, AXIS, Poland)
Data format	Raw, analyzed
Experimental factors	Two series of formulations containing hydrophobized silica nanofiller dispersed in acrylates were prepared and photopolymerized. For SEM analysis composite samples were fractured at room temperature.
Experimental features	Viscosities of monomer/silica mixtures, homogeneity of the filler dispersion in the polymer matrix, and the bulk Poisson׳s ratio of the nanocomposites were determined.
Data source location	Institute of Physics, Poznan University of Technology, Poznan, Poland
Data accessibility	The data are available with this article

**Value of the data**

• Viscosity data can be used to identify differences in viscoelastic behaviour of monomer/silica dispersions. This data is valuable in indicating interactions between hydrophobized surfaces of silica particles and monomers (shear–thinning behaviour).
• SEM data provides information about the homogeneity of the filler dispersion in polymer matrices for the free surface and across the bulk (fractured surface).
• The estimated Poisson׳s ratio can be used for elastic modulus calculation at all length scales (nano-micro-macro).
• The data may be valuable for similar research in future industrial processes.

## 1 Data

In this work, we provide the data obtained for photocurable polyacrylate-based composites filled with hydrophobized nanosilica, both before the polymerization, namely formulation viscosity, and after curing, that is the scanning electron microscopy (SEM) visualization of the their free and fractured surfaces. We estimate the bulk Poisson׳s ratio for these samples, as well. The validation of this study can be found in Ref. [1]. The data presented herein illustrates the effects of monomer-nanosilica interactions on system viscosity, quality of nanosilica dispersion in polymer matrices, and Poisson effect for polymeric nanocomposites with selected filler loadings.

## 2 Experimental design, materials and methods

### 2.1 Materials and samples preparation

2-hydroxyethyl acrylate (HEA) and polyethylene glycol diacrylate (PEGDA, MW 575, *n*=10) were mixed with nanosilica filler (Aerosil R7200). Two acrylate-based series of formulations were thus obtained, containing 0 (neat monomers), 5, 10, 15 and 20 wt% of the silica filler. Specimens for SEM were fractured symmetrically at room temperature and imaged close to the centre of the cutting edge. For details of the samples׳ preparation see Ref. [1].

### 2.2 Methods and data outcome

#### 2.2.1 Viscosity

**Fig. 1:**
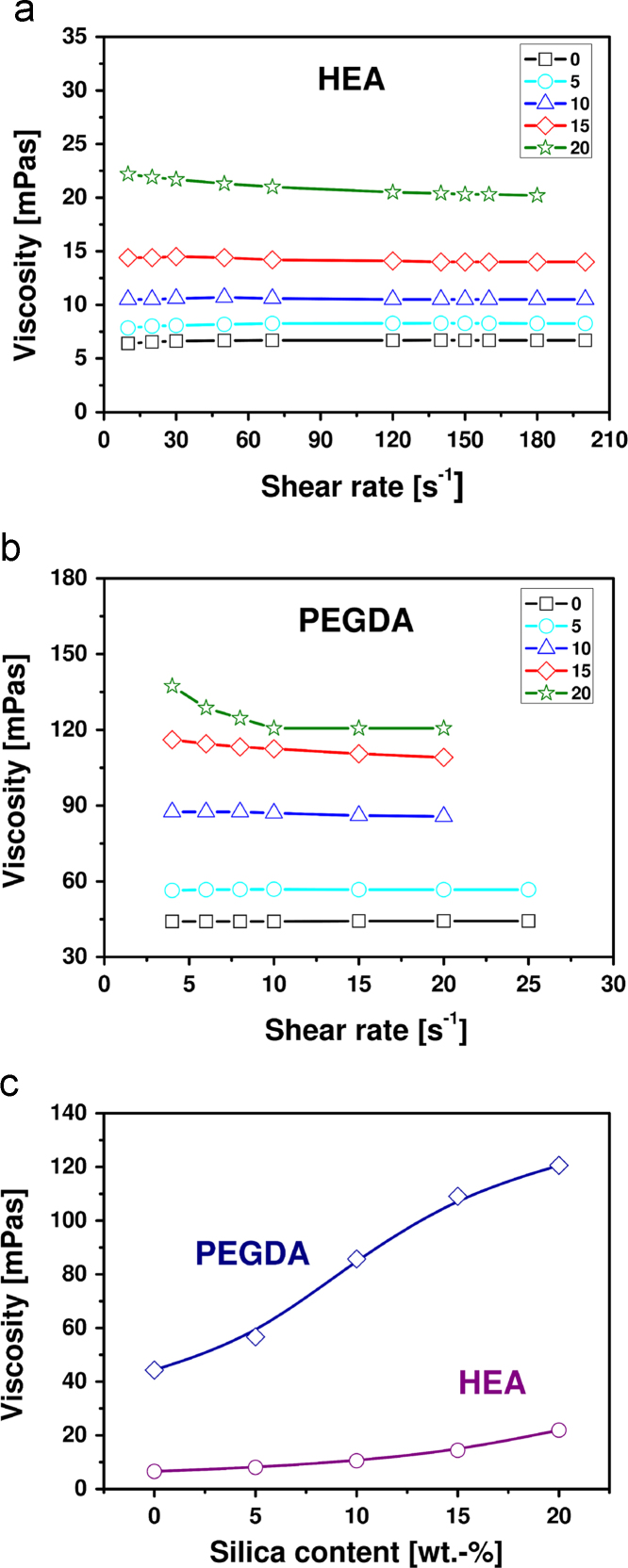
Viscosity of formulations of (a) HEA/silica and (b) PEGDA/silica as a function of the shear rate and (c) silica content (at share rate ~20 s^−1^) at 20 °C. The numbers in the insets indicate the filler content in wt%. The lines are guides to the eye.

The knowledge of formulation viscosity is necessary in studying the composite formation. Fig. 1 shows viscosity of the monomer/silica dispersions as a function of the shear rate (Fig. 1a and b) and the filler content (Fig. 1c) for HEA and PEGDA measured in the cone-and-plate geometry at 20 °C. The viscosity of the composition increases with the silica content. The viscosity of the neat monomers and silica-containing formulations exhibits Newtonian behaviour with some deviations at the silica contents of 15–20 wt% (shear–thinning behaviour). The lack of shear–thinning behaviour indicates the lack of interactions between silica particles having a hydrophobized surface (no network formation between silica particles). A slightly marked shear–thinning effect in the presence of over a dozen percent of silica (15–20 wt%) may result from the fact that although silica Aerosil R7200 is surface modified with methacryloxy groups, it still contains a number of silanol functions which give a possibility to interact (dominant mechanism in the viscoelastic behaviour). The increase of viscosity with increasing silica content is stronger for dispersions in PEGDA than in HEA (stronger interactions of the former with the filler).

#### 2.2.2 Scanning electron microscopy

In order to evaluate dispersion of the filler in the matrices, we analyzed SEM images obtained at low voltage (3 kV) with a working distance of 2–5 mm. Two aspects were our main focus: (1) homogeneity of the dispersion of the filler in the bulk of polymer matrix, and (2) possible differences between the free surface (top part of the sample; formed at the argon-sample interface) and fractured surface (formed by the mechanical cutting; called here as the “bulk” surface). The filler homogenous dispersion is of importance to keep the properties of the bulk uniform. Clustering or agglomeration may result in the weakening of physical properties. Additionally, the interfacial phenomena, derived essentially from the monomer surface tension and particle hydrophobicity, may lead to a particle density gradient (PDG) between the free surface and the bulk in the final photocured nanocomposite. Although this effect is of marginal significance to the macroscopic material properties, it may play a non-negligible role for the surface morphology at micro- and nanoscale, for instance, when investigated with atomic force microscopy (AFM) (see Ref. [1]).

**Fig. 2:**
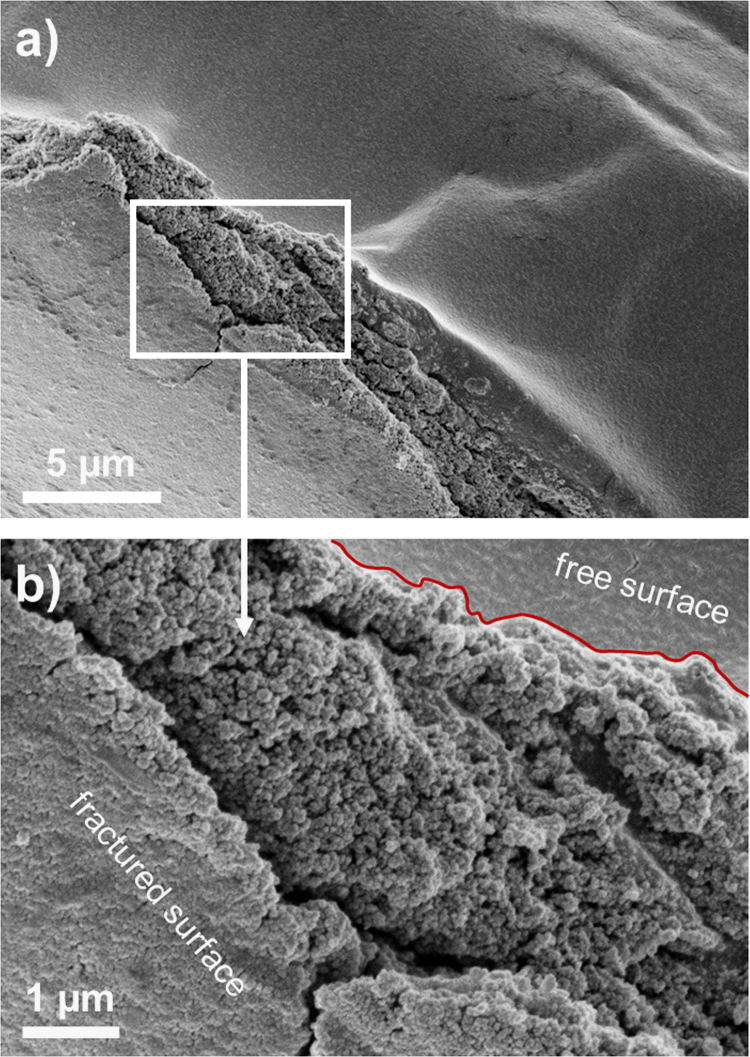
(a) SEM micrograph of nanocomposite containing polyHEA+ 10 wt% of the silica Aerosil R7200 content obtained at the fractural edge. Magnified area – indicated by a white window – is shown in (b). The red line divides area of the free and fractured surfaces.

**Fig. 3:**
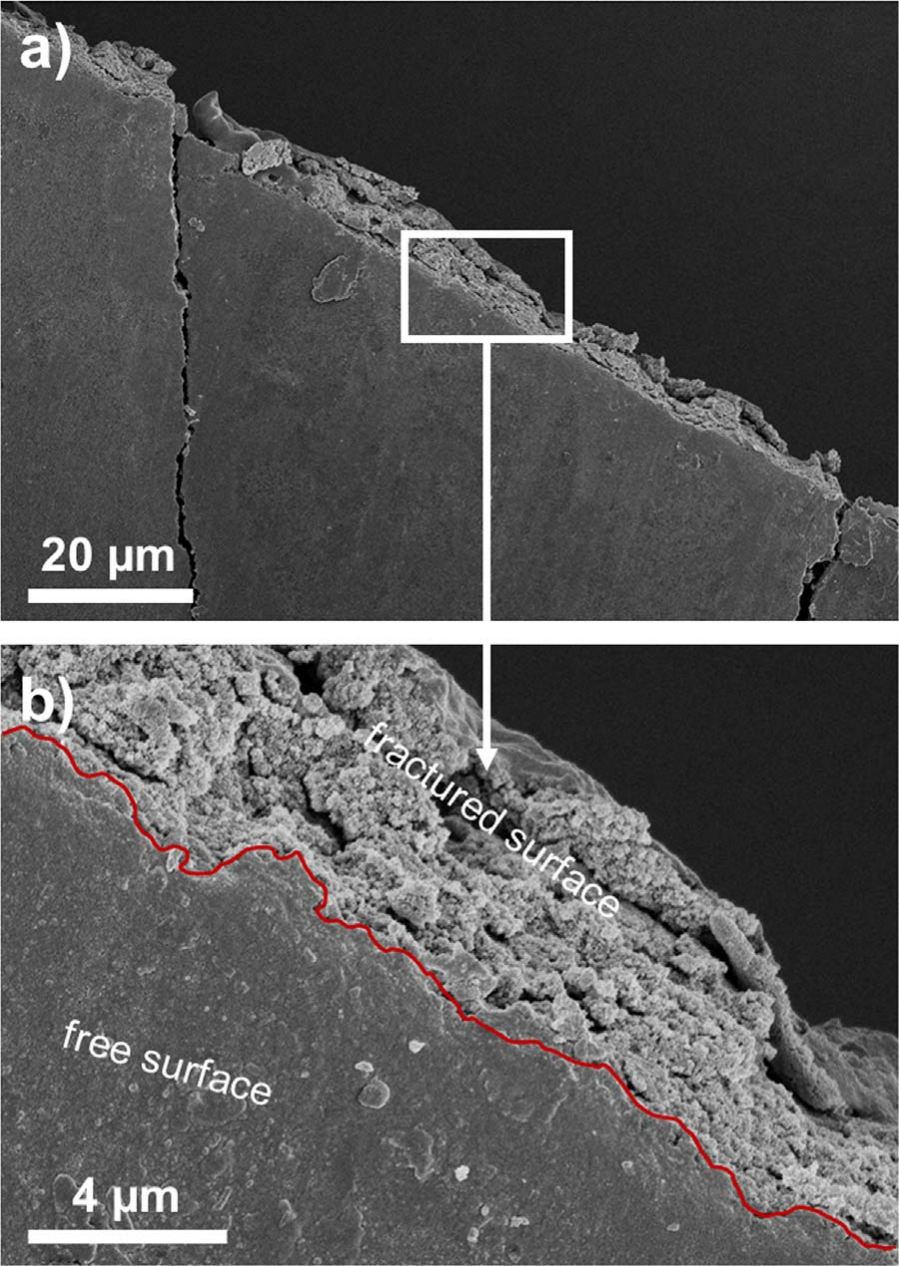
(a) SEM micrograph of nanocomposite containing polyHEA+ 15 wt% of the silica Aerosil R7200 content obtained at the fractural edge. Magnified area – indicated by a white window – is shown in (b). The red line divides area of the free and fractured surfaces.

Fig. 2, Fig. 3 show SEM micrographs of nanocomposite with polyHEA matrix containing 10 and 15 wt% of the silica nanofiller, respectively. The images were taken at the edge of the fractured samples, thus both the distribution over the free surface and dispersion of the filler in the bulk are observed. The interface between the free and fractured surfaces is indicated using a red line. Both surfaces exhibit homogenous distribution of the nanoparticles (regular nanoparticle-print morphology); the polymer matrix is completely permeated with silica nanofiller. Furthermore, a closer look at the cracked area in Fig. 2b indicates no PDG between the free surface and the bulk, at least to be detected by SEM; unperturbed structure continuity of well-defined composite is visible across the fraction. Similar particle assembly was observed for other samples as well (not shown). The addition of the filler (example in Fig. 3) makes the composite more brittle. This is a natural consequence of the reduced volume of the flexible matrix (see glass transition temperatures in Fig. 1 in Ref. [1]) between the nanoparticles.

**Fig. 4:**
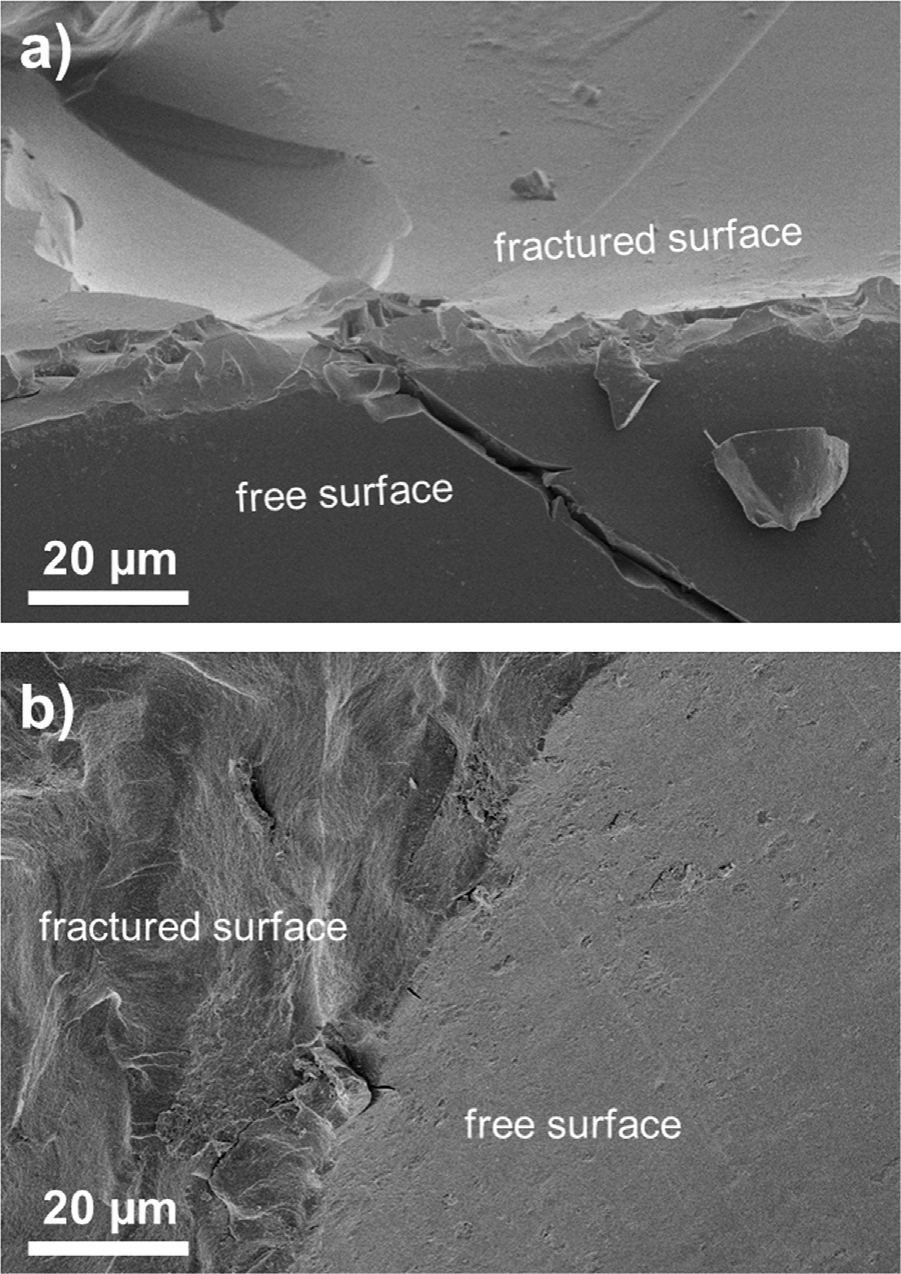
SEM micrographs obtained at the fractural edge: (a) neat polyPEGDA and (b) polyPEGDA+ 15 wt% of the silica Aerosil R7200 content.

Fig. 4 demonstrates differences in surface morphology for neat polyPEGDA sample and its representative filled with 15 wt% of the nanosilica, at the interface of the free and fractured surface. One can see that also in polyPEGDA matrix the filler is homogenously distributed in the bulk. No PDG can be identified either. Thus, the surface morphology, either unveiled on the free or in the bulk surface, for all the investigated samples, are alike.

High resolution SEM and AFM images of the investigated nanocomposites are found in the related article [1].

#### 2.2.3 Bulk Poisson׳s ratio

A complex composite system with a varied content of the nanofiller is characterized by different Poisson׳s ratios. For neat polyHEA and neat polyPEGDA we used the values of 0.40 and 0.45, respectively, for Poisson׳s ratios [2], [3]. The relatively high Poisson׳s ratio for the neat polyPEGDA sample was assumed due to the elastomeric character of its network and, to a large extent, its incompressible behaviours (e.g., for pure elastomers Poisson׳s ratio is 0.49–0.5). To calculate Poisson׳s ratio of composites, we applied the Voigt model (Rule of Mixtures) as the presence of silica notable reduces its value [4], [5]. In the Voigt model the estimation of Poisson׳s ratio is based on the volume weighted average of the phases that are characterized by their individual Poisson׳s ratio. For silica particles we took a typical value of Poisson׳s ratio, i.e. 0.17, and the mass density of 2200 kg m^−^^3^ [6], [7]. The mass density of the neat polymers was obtained by hydrostatic weighing in ethanol; for neat polyHEA and polyPEGDA we obtained 1136 kg m^−3^ and 1193 kg m^−^^3^, respectively. These mass densities share strong similarities with chemical data bases [8]. Table 1 presents calculated Poisson׳s ratios based on the Voigt model.

## Figures and Tables

**Table 1:** Poisson׳s ratio, ν, estimated based on the Voigt model (volume weighted Poisson׳s ratios).

Silica content [wt%]	ν [–]	
	polyHEA	polyPEGDA
5	0.348	0.362
10	0.325	0.347
15	0.311	0.332
20	0.297	0.317
