# Supplementary material for: Dataset for acrylate/silica nanoparticles formulations and photocured composites: Viscosity, filler dispersion and bulk Poisson׳s ratio
Source: Data Brief. 2017 Apr 28;12:528–34. doi: 10.1016/j.dib.2017.04.040 (PMC5425337; doi:10.1016/j.dib.2017.04.040)
Supplement: Supplementary file 1 — Supplementary material [file mmc1.docx]

Dear Journal Office, Data in Brief

Date: 06.03.2017

Title of the manuscript: Dataset for acrylate/silica nanoparticles formulations and photocured composites: viscosity, filler dispersion and bulk Poisson’s ratio

Authors: Hubert Gojzewski, Mariola Sadej, Ewa Andrzejewska, Martyna Kokowska

Authors’ declaration: **Hereby, we inform that we have no conflict of interest to declare.**

Sincerely Yours,
Hubert Gojzewski
on behalf of all authors
